# Inequities in Health Care Services Caused by the Adoption of Digital Health Technologies: Scoping Review

**DOI:** 10.2196/34144

**Published:** 2022-03-21

**Authors:** Rui Yao, Wenli Zhang, Richard Evans, Guang Cao, Tianqi Rui, Lining Shen

**Affiliations:** 1 School of Medicine and Health Management Tongji Medical College Huazhong University of Science & Technology Wuhan China; 2 Faculty of Computer Science Dalhousie University Halifax, NS Canada; 3 Hubei Provincial Research Center for Health Technology Assessment Wuhan China; 4 Institute of Smart Health Huazhong University of Science & Technology Wuhan China

**Keywords:** health inequities, digital health technologies, health care services, socially disadvantaged groups, scoping review, mobile phone

## Abstract

**Background:**

Digital health technologies (ie, the integration of digital technology and health information) aim to increase the efficiency of health care delivery; they are rapidly adapting to health care contexts to provide improved medical services for citizens. However, contrary to expectations, their rapid adoption appears to have led to health inequities, with differences in health conditions or inequality in the distribution of health care resources among different populations.

**Objective:**

This scoping review aims to identify and describe the inequities of health care services brought about by the adoption of digital health technologies. The factors influencing such inequities, as well as the corresponding countermeasures to ensure health equity among different groups of citizens, were also studied.

**Methods:**

Primary studies and literature, including articles and reviews, published in English between 1990 and 2020 were retrieved using appropriate search strategies across the following three electronic databases: Clarivate Analytics’ Web of Science, PubMed, and Scopus. Data management was performed by two authors (RY and WZ) using Thomson Endnote (Clarivate Analytics, Inc), by systematically screening and identifying eligible articles for this study. Any conflicts of opinion were resolved through discussions with the corresponding author. A qualitative descriptive synthesis was performed to determine the outcomes of this scoping review.

**Results:**

A total of 2325 studies were collected during the search process, of which 41 (1.76%) papers were identified for further analysis. The quantity of literature increased until 2016, with a peak in 2020. The United States, the United Kingdom, and Norway ranked among the top 3 countries for publication output. Health inequities caused by the adoption of digital health technologies in health care services can be reflected in the following two dimensions: the inability of citizens to obtain and adopt technology and the different disease outcomes found among citizens under technical intervention measures. The factors that influenced inequities included age, race, region, economy, and education level, together with health conditions and eHealth literacy. Finally, action can be taken to alleviate inequities in the future by government agencies and medical institutions (eg, establishing national health insurance), digital health technology providers (eg, designing high-quality tools), and health care service recipients (eg, developing skills to access digital technologies).

**Conclusions:**

The application of digital health technologies in health care services has caused inequities to some extent. However, existing research has certain limitations. The findings provide a comprehensive starting point for future research, allowing for further investigation into how digital health technologies may influence the unequal distribution of health care services. The interaction between individual subjective factors as well as social support and influencing factors should be included in future studies. Specifically, access to and availability of digital health technologies for socially disadvantaged groups should be of paramount importance.

## Introduction

### Background

An evolution in health care services is occurring across the globe in response to an explosion in readily available digital technologies. The adoption of digital technologies as a means for citizens to access health and social care is accelerating at an unprecedented pace, pushing patient-centered care toward digital health [1]. Many countries and organizations are paying greater attention to digital health, which has seen a sharp increase in the release of health policies and reports, such as the United Kingdom’s *Digital Strategy* published in 2012 [2], the European Union’s *Europe’s Digital Decade* and *Digital Europe Programme* [3], the World Health Organization’s *Draft Global Strategy for Digital Health (2020-2025)* [4], China’s *Outline of Healthy China 2030 Plan* [5], and so on. Digital health refers to the use of readily available information and communication technologies for the following: to provide patients with preventive services, treatment, and education; to promote disease tracking and monitoring; and to enable consumers to participate in health care services [6,7]. Digital health is the integration of digital technology and health information with the aim of increasing the efficiency of health care delivery and improving the health of patients [8]. Digital health technology is the adoption of digital technology in health, with examples being seen in electronic health records, telemedicine or telehealth services, robotics, and eHealth, along with mobile health supported by the use of smartphones, wearables, mobile apps, and various monitoring devices [9-11].

Lupton [12] had said that “Digital health technologies are positioned to enable people to effectively become ‘managers’ of their own health and healthcare.” In our internet-enabled world, the use of digital health technologies is becoming the core of health care delivery. Studies have shown that digital health technology can improve health literacy, enhance patient participation in health care, enable patients to better manage their own health, and improve health care efficiency, especially in patients with chronic diseases [13,14]. Digital health technology interventions, that is, those delivered through digital technologies, such as smartphones and websites [15], can improve health care delivery and contribute to the *triple aim* of health care, that is, better care, better health outcomes, and reduction in medical spending [16]. However, compared with expectations, the rapid development of digital health technologies has led to health inequities.

Health inequities refer to differences in health conditions or the distribution of health care resources among different populations because of social conditions, such as the citizens’ place of birth, growth, life, or work [17]. Although digital health technologies are being adopted rapidly, it is likely that those who do not use the internet or mobile devices regularly or have difficulty in using them, such as older adults, those living in low-income regions, and people in remote areas with poor internet connectivity, will be forgotten [18-20]. This phenomenon not only represents inequities among income, education, and age groups and between the healthiest and least healthy [17] but also represents inequities in access to and availability of technology, which is a continuing barrier to the use of digital health services [21].

The potential of technologies to induce health inequities has been widely recognized [22]. As early as 2016, the World Bank’s Information Industry Report identified that information technology innovations have the potential to lead to new inequities [20]. The report stated that those who are wealthy and better educated are well positioned to take advantage of the internet. However, many global citizens do not have access to the internet. In some regions where women have low socioeconomic status (SES), they are discouraged from going on the web and do not have access to cell phones [20]. In addition, there are still people in parts of the world who are illiterate and do not benefit from access to the internet. Some studies found that mobile health interventions can exacerbate treatment disparities [23,24]. Digital health technology interventions work better for those who are already better off—a situation that can induce inequities. This phenomenon is well established in public health and is referred to as *Intervention-Generated Inequalities* [19]. Socially disadvantaged groups [25] have more challenges in access to and availability of digital health technologies [26], which may lead to more severe health inequities.

### Objectives

From an ethical perspective, health equity is more important than health inequity as the latter can have negative social and economic consequences [27]. The databases of Cochrane, JBI Evidence Synthesis, and PROSPERO (the international prospective register of systematic reviews administered by the University of York’s Centre for Reviews and Dissemination) show that there is no literature review to date on inequities caused by the application of digital health technologies in health care services. The aim of this scoping review is to systematically review and synthesize information on health inequities in health care delivery resulting from digital health technologies and to provide insights for future research and practice. Such a review can provide a better understanding of the health inequities caused by digital health technologies, the influencing factors, and countermeasures and can inform future corresponding policy decision-making to ensure health equities among different citizen groups, and thereby achieve social equity and stability.

## Methods

### Overview

This scoping review used the framework of Arksey and O’Malley [28], which comprises the following five stages: identifying the research question; identifying relevant studies; study selection; data extraction and analysis; and collating, summarizing, and reporting the results. The reporting of the scoping review was guided by the PRISMA-ScR (Preferred Reporting Items for Systematic Reviews and Meta-Analyses extension for Scoping Reviews) checklist; for details of items included in the checklist, please see Multimedia Appendix 1 [29]. In addition, the study was registered in an open science framework and an independent registry. The registration type of this scoping review is open-ended and the registration digital object identifier is 10.17605/OSF.IO/A5R7F.

### Search Strategy

The electronic databases Clarivate Analytics’ Web of Science, PubMed, and Scopus were searched for articles published in English between 1990 (in the late 1990s, the combination of medical care and technology gave birth to a new field called *eHealth* [30]) and 2020. Two coauthors (RY and WZ) developed and performed a Boolean search strategy based on SPIDER (Sample, Phenomenon of Interest, Design, Evaluation, Research) type—a search tool used to identify relevant qualitative and mixed method studies [31]. The search frame included phenomenon of interest (digital technologies, such as *ehealth* or telehealth*), evaluation (inequit*), and research type (primary studies and literature including articles or reviews; see Table 1 for details). Through a review of the keywords that appeared in the title or summary field of each article, a list of literature was retrieved. The title information was then exported to EndNote X9 (Clarivate Analytics, Inc) for evaluation to promote the selection process and collaboration among reviewers.

**Table 1:** Database search strategy.

Database	Search strategy	Number of results
Web of science	TS= (eHealth OR telehealth* OR telemedicine OR mHealth OR mobile health OR health IT OR health information technolog* OR health informat* OR digital health* OR digital health technolog* OR [ICT AND (health* OR healthcare settings OR healthcare delivery OR healthcare service*)] OR [technolog* AND (health* OR healthcare settings OR healthcare delivery OR healthcare service*)]) AND TS= inequit*. Time span=January 1, 1990, to December 31, 2020.	910
PubMed	(1990[Date—Publication]: 2020[Date—Publication]) AND (eHealth[Title/Abstract] OR telehealth*[Title/Abstract] OR telemedicine[Title/Abstract] OR mHealth[Title/Abstract] OR mobile health[Title/Abstract] OR health IT[Title/Abstract] OR health information technolog*[Title/Abstract] OR health informat*[Title/Abstract] OR digital health*[Title/Abstract] OR digital health technolog*[Title/Abstract] OR [ICT(Title/Abstract) AND (health*[Title/Abstract] OR health care settings[Title/Abstract] OR health care delivery[Title/Abstract] OR health care service*[Title/Abstract])] OR [technolog*(Title/Abstract) AND (health*[Title/Abstract] OR health care settings[Title/Abstract] OR health care delivery[Title/Abstract] OR health care service*[Title/Abstract])] OR [telemedicine(MeSH Terms)]) AND (inequit*[Title/Abstract]).	566
Scopus	(TITLE-ABS-KEY[eHealth] OR TITLE-ABS-KEY[telehealth*] OR TITLE-ABS-KEY[telemedicine] OR TITLE-ABS-KEY[mHealth] OR TITLE-ABS-KEY[mobile health] OR TITLE-ABS-KEY[health IT] OR TITLE-ABS-KEY[health information technolog*] OR TITLE-ABS-KEY[health informat*] OR TITLE-ABS-KEY[digital health*] OR TITLE-ABS-KEY[digital health technolog*] OR [TITLE-ABS-KEY(ICT) AND (TITLE-ABS-KEY[health*] OR TITLE-ABS-KEY[healthcare settings] OR TITLE-ABS-KEY[healthcare delivery] OR TITLE-ABS-KEY[healthcare service*])] OR [TITLE-ABS-KEY(technolog*) AND (TITLE-ABS-KEY[health*] OR TITLE-ABS-KEY[healthcare settings] OR TITLE-ABS-KEY[healthcare delivery] OR TITLE-ABS-KEY[healthcare service*])]) AND TITLE-ABS-KEY(inequit*) AND PUBYEAR AFT 1989 AND PUBYEAR BEF 2021.	849

### Inclusion Criteria

Studies were considered eligible if they met the following criteria: (1) published in English between the years 1990 and 2020, (2) either a primary study or literature review, and (3) discussed health inequities related to digital health technology interventions or explored the influencing factors for digital health inducing health inequities or the countermeasures to alleviate health inequities.

### Exclusion Criteria

Studies considered ineligible included those that only related to the following: (1) books or book sections or editorials, commentary, and columns; (2) studies beyond the reach of the full text; (3) public health intervention and policy intervention measures; (4) the design of health technology, service systems, or frameworks to make up for health differences; or (5) studies that did not explore the relationship between digital health technologies and health inequities.

### Study Selection

**Figure 1:**
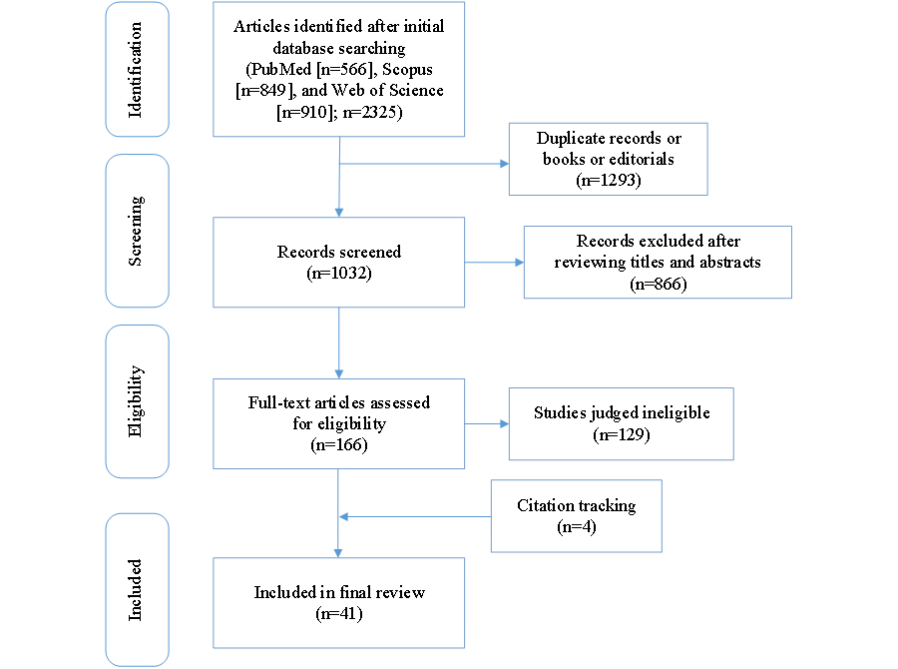
PRISMA-ScR (Preferred Reporting Items for Systematic Reviews and Meta-Analyses extension for Scoping Reviews) procedural flowchart.

After designing the search strategy, inclusion criteria, and exclusion criteria, the literature was reviewed according to the PRISMA-ScR process [29]. A total of 2325 records were collected from the following electronic databases: Clarivate Analytics’ Web of Science, PubMed, and Scopus. To review the relevance of the literature, coauthors RY and WZ screened the titles and abstracts of all remaining records after the removal of duplicates. All full texts were read and analyzed by 2 individual researchers (RY and WZ), and individual data extraction forms were then merged into a single, unifying document that was used for the interpretation and presentation of results. Discrepancies were adjudicated by the corresponding authors. Next, the full article text of the retrieved results was examined by RY and WZ based on the inclusion and exclusion criteria. Of the 2325 papers, 41 (1.76%) papers were identified for the systematic analysis, as illustrated in Figure 1.

### Data Extraction and Analysis

A bespoke data extraction form was used to systematically extract information relevant to the descriptive information. Data were extracted from each article to describe the following: (1) the characteristics of each study (including author, year of publication, publication country, and methods used), (2) the concrete embodiment of the inequity of health care services brought about by the application of digital health technology, (3) the factors contributing to the inequities in health care services resulting from the adoption of digital health technology, and (4) the measures used to mitigate health inequities. Furthermore, a qualitative descriptive synthesis [30] was performed to determine the outcomes of this scoping review.

## Results

### Overview of the Included Studies

**Figure 2:**
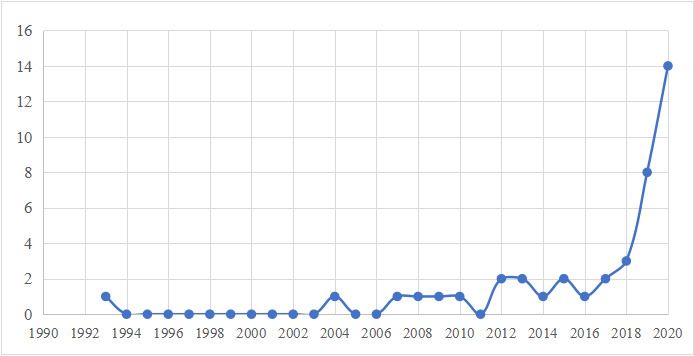
Change in trend of number of publications over time.

Of the 41 papers retained, the earliest publication related to health inequities caused by digital health technologies was published in 1993. Thereafter, the number of relevant publications remained at 0 from 1994-2003, fluctuated between 0 and 2 from 2004-2016, and grew after 2016, reaching a peak of 14 in 2020, as shown in Figure 2.

The United States published the greatest number of papers on the topic, accounting for 46% (19/41), followed by the United Kingdom (8/41, 20%), and Norway (3/41, 7%), whereas other countries published only 1 or 2 papers, as shown in Table 2. Of the 41 records, 6 (15%) were literature reviews, 17 (41%) records used a quantitative approach, 15 (37%) records used a qualitative approach, and 3 (7%) records used mixed methods. Specific information on the included studies is presented in Table 3.

**Table 2:** Statistics of literature publications in different countries (N=41).

Country	Number of literatures, n (%)
Australia	1 (2)
Bangladesh	1 (2)
Canada	2 (5)
Indonesia	1 (2)
Israel	1 (2)
Italy	2 (5)
Korea	1 (2)
Netherlands	1 (2)
Norway	3 (7)
Switzerland	1 (2)
United Kingdom	8 (20)
United States	19 (46)

**Table 3:** Overview of included studies.

Authors	Year	Country	Research method	Manifestations of health inequities	Influencing factors of health inequities	Countermeasures for health inequities
Yang [32]	1993	Korea	Quantitative approach	Access to health care resource: medical insurance access	Income	Countermeasures of government agencies: establish national health insurance
Steiger et al [33]	2004	Switzerland	Qualitative approach	N/A^a^	Age and SES^b^	Countermeasures of technology providers: design high-quality websites
Viswanath et al [34]	2007	United States	Qualitative approach	Access to health care resource: technical access and availability	SES	Countermeasures of government agencies: structural adjustment at the policy system level
Kim et al [35]	2009	United States	Quantitative approach	Access to health care resource: technology acquisition	Economy, age, and eHealth literacy	Countermeasures of government agencies: free PCs, internet connections, and help from nursing students and accommodation staff
Andreassen et al [36]	2010	Norway	Qualitative approach	Health and disease outcome: disease mortality	SES and health literacy	N/A
Goldberg [37]	2012	United States	Qualitative approach	Health and disease outcome: disease risk	SES	Countermeasures of government agencies: give priority to people with diseases
Jones [38]	2013	United Kingdom	Quantitative approach	N/A	Health condition and economic barriers	Countermeasures of government agencies and health care service receivers: provide technical support, medical institutions take actions or volunteer services
Jennings et al [23]	2013	United States	Overview	Access to health care resource: technology acquisition	N/A	N/A
McAuley [18]	2014	United Kingdom	Combination of qualitative and quantitative	Access to health care resource: technology acquisition; health and disease outcome: increased risk of disease	SES and age	Countermeasures of health care service receivers: develop information and skills for access to digital technology
Albright et al [39]	2015	United States	Quantitative approach	Access to health care resource: technology acquisition	Age	N/A
Matteucci [40]	2015	Italy	Qualitative approach	Health and disease outcome: disease risk	Health condition	Countermeasures of government agencies: role of community and health professionals
Mierlo et al [41]	2016	United Kingdom	Quantitative approach	Access to health care resource: digital participation	N/A	N/A
Latulippe et al [42]	2017	Canada	Overview	Health and disease outcome: disease prevalence, mortality; access to health care resource: technology acquisition	eHealth literacy, age, SES, rural, and sexual orientation	Countermeasures of technology providers: universal eHealth care tool and technology design
Hosseinpoor et al [43]	2017	Indonesia	Qualitative approach	Health and disease outcome: Health outcomes; access to health care resource: technical services	N/A	Countermeasures of government agencies: health inequities monitoring embedded in national health information system
Veinot et al [19]	2018	United States	Qualitative approach	N/A	Education level and health literacy	Countermeasures of government agencies and technology providers: invest in resources and time or technical design that is easy to understand and health professionals publish relevant policies
Bol et al [44]	2018	Netherlands	Quantitative approach	Access to health care resource: digital health technologies	Age, education, and eHealth literacy	Countermeasures of government agencies: develop more detailed strategies to bridge the digital divide
Weiss et al [45]	2018	Italy	Overview	Health and disease outcome: disease morbidity and mortality	SES, health condition, age, and race	N/A
Gann [46]	2019	United Kingdom	Qualitative approach	Access to health care resource: technical services	Age, SES, and rural	Countermeasures of health care service receivers: the role of libraries in providing resources
Toscos et al [47]	2019	United States	Quantitative approach	Access to health care resource: digital health technologies	Age and SES	Countermeasures of government agencies and medical institutions: health science researchers should consider the population with needs and insufficient coverage
Sherman et al [1]	2019	United States	Qualitative approach	Health and disease outcome: health outcomes	Race	N/A
Parker et al [48]	2019	United States	Quantitative approach	Health and disease outcome: health outcomes	N/A	N/A
Hansen et al [49]	2019	Norway	Quantitative approach	Access to health care resource: technical services; health and disease outcome: disease morbidity and mortality	SES, age, and health condition	N/A
Baum et al [50]	2012	Australia	Qualitative approach	Access to health care resource: technical services	SES and health literacy	Countermeasures of government agencies: health promotors must understand and explain the complex interaction among digital literacy, health literacy, and basic literacy
Banerjee [51]	2019	United Kingdom	Qualitative approach	N/A	Race	Countermeasures of government agencies and technology providers: health professionals should receive better training in the evidence and use of DHI^c^; the participant should be consulted during the design and implementation phase of the technology
Rich et al [52]	2019	United Kingdom	Qualitative approach	N/A	Economy	Policy documents should focus on inequities
Ahmed et al [53]	2020	Bangladesh	Combination of qualitative and quantitative	Access to health care resource: technical services	Age, SES, and digital health awareness and skills	Countermeasures of health care service receivers: raising public awareness and political incentives
Glied et al [54]	2008	United States	Quantitative approach	Health and disease outcome: disease mortality	Education	N/A
Fujioka et al [55]	2020	Canada	Overview	Health and disease outcome: health outcomes	Race, income, and health condition	N/A
Khilnani et al [56]	2020	United States	Qualitative approach	Health and disease outcome: health outcomes	Age, SES, rural, and digital resources	N/A
DeGuzman et al [57]	2020	United States	Quantitative approach	Access to health care resource: technical services	Rural and broadband coverage	Countermeasures of health care service receivers: public libraries provide equipment and hardware support
Gann [58]	2020	United Kingdom	Qualitative approach	Health and disease outcome: health outcomes	Economy and age	Countermeasures of government agencies: encourage enterprises to donate tablets, smartphones, and laptops, and provided tablet devices to hospitals, nursing homes, and hospice care institutions
Bommakanti et al [59]	2020	United States	Quantitative approach	N/A	Age and SES	N/A
Weiss et al [60]	2020	Norway	Quantitative approach	Health and disease outcome: health outcomes	SES	N/A
Nittas et al [24]	2020	United Kingdom	Overview	Access to health care resource: access and availability of technology	Race, SES, and place of residence	N/A
Karri et al [61]	2020	United States	Quantitative approach	Access to health care resource: technical services	SES, race, age, and health condition	N/A
Jaffe et al [62]	2020	Israel	Quantitative approach	N/A	Age and rural	N/A
DeGuzman et al [63]	2020	United States	Combination of qualitative and quantitative	Access to health care resource: digital health technologies	Digital literacy and skills and provision of broadband facilities	N/A
Sun et al [64]	2020	United States	Qualitative approach	Access to health care resource: technical services	Digital literacy	Countermeasures of government agencies: strengthen the national digital health strategy and the governance of human rights–oriented digital health technologies at the national level
Ukoha et al [65]	2019	United States	Quantitative approach	Health and disease outcome: health outcomes and care	Socioeconomic factors, age, and race	N/A
Hamideh et al [66]	2020	United States	Review	N/A	Age, race, income, rural, health condition, and health literacy	N/A
Erhunmwunsee et al [67]	2020	United States	Quantitative approach	Health and disease outcome: treatment results	Income level, rural location of the hospital and insurance status	N/A

^a^N/A: not available.

^b^SES: socioeconomic status.

^c^DHI: digital health intervention.

### Inequities Caused by Digital Health Technologies

Health inequities were reflected in 2 aspects. The first was access to and availability of digital health technologies by different social groups, with 19 (46%) of the 41 studies describing unfair distribution. Among these 19 studies, 9 (47%) studies referred to a lack of network infrastructure (eg, internet broadband access, satellite towers, and power) and the availability of smartphones or computers when using digital health technologies [32,39,41,43,47,53,57,63,64]. A study pointed out that access to home-based telemedicine was inequitably distributed in the United States owing to the limited reach of fixed broadband in rural areas [57]. Of the 41 studies, 12 (29%) studies reported an unfair phenomenon of digital exclusion [19,23,24,34,35,41,44,46,49,50,61,64]. A study described how, without proper planning and safeguards, digital health technologies can contribute to expanding health inequity, widening the *digital divide* that separates those who can from those who cannot access such interventions [64].

The remaining (17/41, 41%) studies discussed the related health outcomes caused by the lack of or limited access to digital health technologies [1,18,36,37,40,42,43,45,48,49,54-56,58,60,65,67]. Researchers have reported different health outcomes following the introduction of digital health technology interventions, including disease incidence rates and mortality, with particular attention paid to chronic diseases and Black groups [49,56,58,60]. For example, the average blood sugar level of diabetic patients who use innovative health technologies generally drops [49], and the use of robotic lobectomy is limited by sociodemographic factors, leading to significant treatment differences in patients with lung cancer [67].

### Factors Leading to Health Inequities

Most (37/41, 90%) papers reported the factors that influence health inequities brought about by digital health services. Of these 37 studies, 18 (49%) studies reported the important role of age in determining whether patients use or do not use digital health technologies, especially among the older adults [18,33,35,39,42,44-47,49,53,56,58,59,61,62,65,66]. Race and ethnicity have long been considered as one of the *causes of the causes* of health inequities [51]; the acknowledged health benefits of eHealth or mobile health initiatives have seen limited application among Black communities [1,24,42,45,51,55,61,65,66]. In addition, some studies (8/41, 20%) reported that citizens living in rural areas were affected by poor access to and availability of digital health technologies resulting from limited internet broadband coverage [24,42,45,46,56,57,62,66]. In rural areas with multiple health barriers, the unequal distribution of health information is more complex and, therefore, may exacerbate rather than narrow the gap [68]. Furthermore, SES (eg, education and income) [69] was also discussed. Some (18/41, 44%) studies have reported differences in the acquisition and adoption of digital health technologies by different groups of citizens based on a combination of income and education [18,24,33,34,36,37,42,44,46,47,49,50,53,56,59-61,64], whereas others (13/41, 32%) have independently explored the 2 dimensions of income [32,35,38,52,55,58,65-67] and educational attainment [19,44,54,62].

In addition to the above factors, poor health conditions have been observed to hinder access to digital health technologies at a physical level [38,40,45,49,55,61] or a lack of confidence in health advice and health decision-making at a psychological level [38,42]. A literature review found that some people may have difficulty learning to use the internet because of visual or hearing impairment, arthritis, or hand movement difficulties [38]. Studies (11/41, 27%) have also pointed out that the so-called low eHealth literacy (defined as “the ability to seek, find, understand, and appraise health information from electronic sources and apply knowledge gained to addressing or solving a health problem” [70]), including a lack of awareness of digital health services, literacy, and skills, as well as the understanding of the operation mode and availability of digital health, may reduce acceptance of digital health technologies [20,35,36,38,41,42,50,53,63,64,66].

### Countermeasures Lessening Health Inequities

#### Measures of Government Agencies and Medical Institutions

Government agencies and medical institutions are required to alleviate the inequities in digital health technology interventions. This study identified that human rights–oriented digital health technologies should be strengthened at the national level [64] and new research trajectories for policy documents must explore how culture, practice, and power relationships influence access, availability, and engagement with digital health technologies [34,52]. Furthermore, those who develop and apply these interventions, including policymakers and public health professionals, should have a better understanding of whether, how, and under what circumstances digital health technology interventions can overcome inequities and realize their potential [19,40,51,61]. Studies (2/41, 5%) have also recommended that the monitoring of health inequities be embedded within national health information systems and suggested that hybrid web-based and offline interventions can ameliorate health inequities [20,43].

Studies (4/41, 10%) reported that government agencies and medical institutions should provide help with resources, whereby government agencies could establish universal health insurance [32], provide free PC and internet connection help [35,38], and provide limited resources to treat disabled populations, such as those with brain injuries [37]. At the same time, government agencies should ensure increased public funding input [65] and encourage companies to donate tablets, smartphones, and laptops to hospitals, care homes, and hospice facilities [58] to make the use of services more accessible to socially disadvantaged groups, such as families who were not originally connected to the network [47].

#### Actions of Digital Health Technology Providers

Digital health services are inseparable from the design and development of targeted digital health technology. A study in Switzerland [33] proposed the adoption of high-quality internet portals with hashtags to promote autonomous learning and use among citizens. A study pointed out that most digital health technologies were not designed for socially disadvantaged groups, such as older adults or those with limited health literacy skills [47]. Therefore, the individual needs of groups should be carefully considered, including those of groups that need to be consulted during the design and implementation stage [42,51]; resources and time should be invested in the design of easy-to-understand language [19] and bespoke digital health tools should be created and popularized to maximize acceptability [51].

#### Recommended Measures for Health Care Service Receivers

The eHealth literacy of health care service receivers is an important factor hindering access to digital health services. Addressing health inequities depends on increasing public awareness [53]. Some studies (5/41, 12%) suggested that relevant technical tools and volunteers can be provided [1,38] to develop patients’ confidence and skills in digital technology access and improve their eHealth literacy [18,38,50,58]. In addition, some studies (2/41, 5%) reported on the important role of libraries. As a special public space, libraries have unique benefits, including auxiliary digital access, health information resources and services [46,57], and the voluntary services they provide, which can help patients improve their health literacy [46].

## Discussion

### Principal Findings

This scoping review identifies and describes the health inequities in health care services brought about by the adoption of digital health technologies. The evolution of publications over time and their distribution by country in the included literature reflects the concerns of related researchers to some extent. The rate at which the related literature has increased over time is consistent with the developing trend in health information technology. The included literature that has been published from 12 countries indicate the specific prevalence of health inequities on a global scale. Among them, the United States and the United Kingdom have published the most papers, mainly because they were the first to invest heavily in digital health information. Accordingly, health inequities caused by digital health technologies have been quickly noticed and studied.

The health inequities caused by digital health technologies highlight that not all citizens have equal access to and use of interventions, resulting in different health outcomes for the population. From our analysis of the studies investigated, we identified that these indicators, including age, race, SES, health conditions, eHealth literacy, and geographic location, all affect health inequity among groups. First, most theoretical frameworks that are widely used to understand the adoption of new technologies by users, such as *the technology acceptance model*, *the extended unified theory of acceptance and use of technology*, and the diffusion of innovations theory, have demonstrated potential individual differences by considering factors such as age and race. Regarding *age*, we found that older patients had less ownership, which was consistent with other studies [59,71]. During the COVID-19 pandemic, mobile smart devices have been the most effective way for people to seek medical treatment. The older adults may be unable to use mobile devices to register health codes or even for medical service preregistration. However, their long-term demand for medicines is higher [46], reflecting the gap in the use of digital health technologies among different age groups. Rogers [72], in his book *Diffusion of Innovations*, proposed that the older adults belong to laggards; they are relatively conservative when they encounter technological innovation and they feel skeptical and cautious about new things, which may affect their health. Meanwhile, in terms of *gender*, previous studies have reported a general trend that women are more involved in health issues, eHealth, and social media [49]; however, there are also studies that report that women in Africa are the least likely to use digital health technologies worldwide [46]. Moreover, previous studies have found that gender and some ethnic differences in internet use may have disappeared among the general population [73,74] but differences will still expand with age. Health inequities have gradually become a social issue that has received attention from government decision-making departments and related institutions. The National Institutes of Health added a health information section to its official website, which includes links to many health care websites, such as child and adolescent health, men’s health, women’s health, minority health, and older adult health, to provide users and inquirers with the ability to obtain web-based health information.

Second, health inequities caused by *socioeconomic status* are prevalent throughout society, and digital health technologies are accessed to a greater degree by individuals with higher social standing. Link [75] referred to SES as a fundamental factor in health inequities. Social causality provides a theoretical explanation for health inequities and maintains that SES, such as education and income, are an important cause of health inequities. The *knowledge gap* hypothesis [76] holds that as the mass media increasingly disseminate information to society; higher SES groups access information at a faster rate than lower SES groups, so the knowledge gap between these groups will expand. On the basis of existing studies, the main reasons for health inequities caused by SES are as follows: (1) increased income can improve living conditions and access to digital health technologies and (2) a good educational level provides advantages in the adoption of digital health technologies [77]. As early as 20 years ago, Gordon Brown, the United Kingdom’s Chancellor of the Exchequer, announced a plan to *shrink the digital divide*, proposing the provision of free internet access for poor communities, free information technology training, and 100,000 second-hand computers for low-income families. As early as 2010, the United States enacted the *Affordable Care Act*, which promoted access to and availability of health care information resource services by inquirers, especially those that were uninsured and low-income [78].

Third, as health literacy in the digital era may be considered a prerequisite for solving health problems in web-based environments [44], it requires people to better understand mobile health devices such as mobile health apps and how to obtain health information through the internet. In addition, the level of health literacy in the digital era determines the severity of health inequities to a large extent and the level of health literacy in the digital era is not determined by individuals alone. Compared with individual-level behavioral interventions, policy- and system-level interventions often have a greater impact on a population’s health literacy in the digital era [34,79]. In February 2000, the US government released the report *From the Digital Divide to Digital Opportunity*, pointing out that the importance of bridging the digital divide lies in the popularization of professional technology, skills training, and the practicality of network content.

As a result, it can be found that it is necessary for stakeholders to take some measures to jointly build patient-centric digital health technology services to reduce inequities, promoting greater patient access to and use of high-quality digital health technology services. First, *policymakers and medical decision-makers* should promote digital health technologies to poverty-stricken areas and provide low-income patients with free and high-quality health information resources and services, as appropriate [56]. Second, *medical institutions* should simplify the web-based service process and create channels for family members, relatives, friends, and physicians to make appointments. Manual service windows, such as registration, payment, and printing test results, should be reserved to deliver a combination of web-based and offline medical services [24]. Third, *related institutions* should increase investment in education and train individuals in health literacy or health information literacy to improve health outcomes and reduce health inequities [44]. Meanwhile, it is suggested that *relevant departments* should promote and encourage the older adults, the poor, the poorly educated, and other socially disadvantaged groups to use the internet and enrich and standardize the health information and knowledge of the internet to improve their eHealth literacy [18,38]. In view of the difficulties in the application of the internet in daily life, industry training institutions and experts should be organized to carry out special training to improve their operational ability with digital health technologies. Finally, when designing tools, *health care service providers* must participate in the development of assistive technologies, simplifying operations to help individuals who are older, have poor electronic literacy, or are poorly educated to overcome difficulties in using the internet and consider the patient’s reading and writing skills and major languages, as well as the ways and facilities used [80]. In addition, *guide manufacturers* should design and produce special easy-to-use manuals and video tutorials for the functions of products commonly used by the older adults [51].

### Research Gaps

We found that the literature included in this study still had certain research gaps. First, from the perspective of research methods, most of the included literature was based on statistical analysis and a qualitative approach to confirm the influence of various factors such as age on health inequities. Future research can use machine learning, deep learning, and other methods to explore the impact of more indicators on health inequality, dig deeper into the incidence relation between indicators, and then analyze the causal relationship of related indicators to propose more targeted countermeasures.

Second, in terms of influencing factors, scholars have studied objective factors such as age, race, rural residence, health conditions, and eHealth literacy. However, subjective factors, such as an individual’s willingness to use digital health technologies and attitudes toward the internet, have not been studied. Age and education also influence eHealth literacy but the literature we have included does not study the moderating effect of age, education, and other factors on the relationship between eHealth literacy and health inequities. Therefore, future research could include subjective factors and the social support received by individuals while considering the moderating effects of multiple factors.

Finally, from the strategy perspective, the included literature showed that no more innovative technologies were applied to socially disadvantaged groups. In the new digital health era, people are demanding more high-quality and personalized health care services. In the future, services should be provided to different socially disadvantaged groups based on their population classification. The integration of multiple digital health technologies, such as internet multiparty voice call technology and virtual reality, could be applied to disease diagnosis and treatment, virtual reality in surgery, telemedicine, and health management monitoring to assist individuals in obtaining better health care services.

### Limitations

This study had several limitations. First, we only searched the literature in the three databases of Clarivate Analytics’ Web of Science, PubMed, and Scopus, which may have resulted in the related literature not being retrieved. Second, owing to the large differences in the types of literature studies and outcome indicators included in the study, only 17 (41%) of the 41 included articles discussed related health consequences, such as morbidity and mortality, caused by digital health technologies. Therefore, we failed to perform a meta-analysis of quantifiable outcome measures. Third, we aimed to reduce publication bias, and although our inclusion criteria were broad, our search was limited to articles written only in English. Finally, the decision to exclude gray literature, including books and reports, might have led to the exclusion of relevant literature that could possibly have been used to widen or further support the perspectives presented in our results. Nevertheless, as gray literature includes reports and documents often drafted by political or special-interest organizations, it is more difficult to assess underlying biases, which may negatively add bias to our results.

### Conclusions

This scoping review investigates the existing literature on inequities in health care services caused by the adoption of digital health technologies. We identified health inequities brought about by digital health technologies, related influencing factors, and countermeasures against the inequities. The results of the review show that health inequities caused by the adoption of digital health technologies in health care services can be reflected in the following two dimensions: the inability of the population to obtain and adopt technology (eg, no access to technology) and the different disease outcomes among populations under technical intervention measures (eg, disease mortality). In addition, this study found that factors (including age, race, region, economy, and education level), health conditions, and eHealth literacy had an impact on the inequities. Government agencies, medical institutions, digital technology providers, and health care service receivers need to initiate relevant actions to reduce these inequities. Considering the increasing number of health technology interventions provided by mobile technologies, digital health plans that may bring inequities should be implemented and evaluated more carefully. Meanwhile, all parties should pay attention to the impact of individual subjective factors, social support, and the interactions of different factors. The results of this review can help socially disadvantaged groups acquire and use digital health technologies more efficiently, thereby ensuring health equity among different groups of citizens and achieving social equity and social stability.

## Appendix

Multimedia Appendix 1 The details of PRISMA-ScR (Preferred Reporting Items for Systematic Reviews and Meta-Analyses extension for Scoping Reviews) checklist.

## Abbreviations

PRISMA-ScR: Preferred Reporting Items for Systematic Reviews and Meta-Analyses extension for Scoping Reviews
SES: socioeconomic status
SPIDER: Sample, Phenomenon of Interest, Design, Evaluation, Research

## References

[1] Sherman LD, Grande SW (2019). Building better clinical relationships with patients: an argument for digital health solutions with black men. Health Serv Insights.

[2] (2012). Digital strategy: leading the culture change in health and care. Department of Health.

[3] (2021). State of the union: commission proposes a path to the digital decade to deliver the EU's digital transformation by 2030. European Commission.

[4] (2019). Draft global strategy for digital health (2020-2025). World Health Organization.

[5] (2016). Outline of healthy China 2030 Plan. China Government Network.

[6] Rodriguez JA, Clark CR, Bates DW (2020). Digital health equity as a necessity in the 21st Century Cures Act era. JAMA.

[7] (2019). WHO guideline: recommendations on digital interventions for health system strengthening. World Health Organization.

[8] Sharma A, Harrington RA, McClellan MB, Turakhia MP, Eapen ZJ, Steinhubl S, Mault JR, Majmudar MD, Roessig L, Chandross KJ, Green EM, Patel B, Hamer A, Olgin J, Rumsfeld JS, Roe MT, Peterson ED (2018). Using digital health technology to better generate evidence and deliver evidence-based care. J Am Coll Cardiol.

[9] Seckman C, Van de Castle B (2021). Understanding digital health technologies using mind maps. J Nurs Scholarsh.

[10] Scott BK, Miller GT, Fonda SJ, Yeaw RE, Gaudaen JC, Pavliscsak HH, Quinn MT, Pamplin JC (2020). Advanced digital health technologies for covid-19 and future emergencies. Telemed J E Health.

[11] (2019). Evidence standards framework for digital health technologies: user guide. National Institute for Health and Care Excellence (NICE).

[12] Lupton D, Davis JE, Gonzalez AM (2016). Digitized health promotion: personal responsibility for health in the web 2.0 era. To fix or to heal: patient care, public health, and the limits of biomedicine.

[13] Jones SS, Rudin RS, Perry T, Shekelle PG (2014). Health information technology: an updated systematic review with a focus on meaningful use. Ann Intern Med.

[14] Chaudhry B, Wang J, Wu S, Maglione M, Mojica W, Roth E, Morton SC, Shekelle PG (2006). Systematic review: impact of health information technology on quality, efficiency, and costs of medical care. Ann Intern Med.

[15] Murray E, Hekler EB, Andersson G, Collins LM, Doherty A, Hollis C, Rivera DE, West R, Wyatt JC (2016). Evaluating digital health interventions: key questions and approaches. Am J Prev Med.

[16] Berwick DM, Nolan TW, Whittington J (2008). The triple aim: care, health, and cost. Health Aff (Millwood).

[17] Klein R, Huang D (2010). Defining and measuring disparities, inequities, and inequalities in the healthy people initiative. Centers for Disease Control and Prevention.

[18] McAuley A (2014). Digital health interventions: widening access or widening inequalities?. Public Health.

[19] Veinot TC, Mitchell H, Ancker JS (2018). Good intentions are not enough: how informatics interventions can worsen inequality. J Am Med Inform Assoc.

[20] (2016). The World Bank report says innovation in information technology could lead to new inequities. Global Network.

[21] Krouse HJ (2020). COVID-19 and the widening gap in health inequity. Otolaryngol Head Neck Surg.

[22] Shankardass K, Lofters A, Kirst M, Quiñonez C (2012). Public awareness of income-related health inequalities in Ontario, Canada. Int J Equity Health.

[23] Jennings L, Gagliardi L (2013). Influence of mHealth interventions on gender relations in developing countries: a systematic literature review. Int J Equity Health.

[24] Nittas V, Ameli V, Little M, Humphreys DK (2020). Exploring the equity impact of mobile health-based human immunodeficiency virus interventions: a systematic review of reviews and evidence synthesis. Digit Health.

[25] (2013). Disadvantaged groups. European Institute for Gender Equality.

[26] Turnbull S, Lucas PJ, Hay AD, Cabral C (2020). Digital health interventions for people with type 2 diabetes to develop self-care expertise, adapt to identity changes, and influence other's perception: qualitative study. J Med Internet Res.

[27] Baciu A, Negussie Y, Geller A, Weinstein JN, National Academies of Sciences, Engineering, and Medicine, Health and Medicine Division, Board on Population Health and Public Health Practice, Committee on Community-Based Solutions to Promote Health Equity in the United States (2017). Communities in action: pathways to health equity.

[28] Arksey H, O'Malley L (2005). Scoping studies: towards a methodological framework. Int J Soc Res Methodol.

[29] Tricco AC, Lillie E, Zarin W, O'Brien KK, Colquhoun H, Levac D, Moher D, Peters MD, Horsley T, Weeks L, Hempel S, Akl EA, Chang C, McGowan J, Stewart L, Hartling L, Aldcroft A, Wilson MG, Garritty C, Lewin S, Godfrey CM, Macdonald MT, Langlois EV, Soares-Weiser K, Moriarty J, Clifford T, Tunçalp Ö, Straus SE (2018). PRISMA extension for scoping reviews (PRISMA-ScR): checklist and explanation. Ann Intern Med.

[30] André A, André A (2019). The information technology revolution in health care. Digital medicine.

[31] Cooke A, Smith D, Booth A (2012). Beyond PICO: the SPIDER tool for qualitative evidence synthesis. Qual Health Res.

[32] Yang BM (1993). Medical technology and inequity in health care: the case of Korea. Health Policy Plan.

[33] Steiger TS, Duetz Schmucki M (2004). Health information on the internet: an issue of social inequality in public health. Soz Praventivmed.

[34] Viswanath K, Kreuter MW (2007). Health disparities, communication inequalities, and eHealth. Am J Prev Med.

[35] Kim EH, Stolyar A, Lober WB, Herbaugh AL, Shinstrom SE, Zierler BK, Soh CB, Kim Y (2009). Challenges to using an electronic personal health record by a low-income elderly population. J Med Internet Res.

[36] Andreassen HK, Dyb K (2010). Differences and inequalities in health: empirical reflections on telemedicine and politics. Inf Commun Soc.

[37] Goldberg DS (2012). Justice, population health, and deep brain stimulation: the interplay of inequities and novel health technologies. AJOB Neurosci.

[38] Jones R (2013). Development of a questionnaire and cross-sectional survey of patient eHealth readiness and eHealth inequalities. Med 2 0.

[39] Albright KC, Boehme AK, Mullen MT, Wu TC, Branas CC, Grotta JC, Savitz SI, Wolff C, Sen B, Carr BG (2015). The effect of telemedicine on access to acute stroke care in Texas: the story of age inequalities. Stroke Res Treat.

[40] Matteucci I (2015). Social determinants of health inequalities: moving toward a socio-constructivist model supported by information and communication technologies. Global Bioethics.

[41] van Mierlo T, Hyatt D, Ching AT (2016). Employing the Gini coefficient to measure participation inequality in treatment-focused digital health social networks. Netw Model Anal Health Inform Bioinform.

[42] Latulippe K, Hamel C, Giroux D (2017). Social health inequalities and eHealth: a literature review with qualitative synthesis of theoretical and empirical studies. J Med Internet Res.

[43] Hosseinpoor AR, Nambiar D, Tawilah J, Schlotheuber A, Briot B, Bateman M, Davey T, Kusumawardani N, Myint T, Nuryetty MT, Prasetyo S, Floranita R, Suparmi (2018). Capacity building for health inequality monitoring in Indonesia: enhancing the equity orientation of country health information system. Glob Health Action.

[44] Bol N, Helberger N, Weert JC (2018). Differences in mobile health app use: a source of new digital inequalities?. Inf Soc.

[45] Weiss D, Rydland HT, Øversveen E, Jensen MR, Solhaug S, Krokstad S (2018). Innovative technologies and social inequalities in health: a scoping review of the literature. PLoS One.

[46] Gann B (2019). Transforming lives: combating digital health inequality. IFLA J.

[47] Toscos T, Drouin M, Pater J, Flanagan M, Pfafman R, Mirro MJ (2019). Selection biases in technology-based intervention research: patients' technology use relates to both demographic and health-related inequities. J Am Med Inform Assoc.

[48] Qureshi S, Xiong J, Deitenbeck B (2019). The effect of mobile health and social inequalities on human development and health outcomes: mhealth for health equity. Proceedings of the 52nd Hawaii International Conference on System Sciences.

[49] Hansen AH, Bradway M, Broz J, Claudi T, Henriksen Ø, Wangberg SC, Årsand E (2019). Inequalities in the use of eHealth between socioeconomic groups among patients with type 1 and type 2 diabetes: cross-sectional study. J Med Internet Res.

[50] Baum F, Newman L, Biedrzycki K (2014). Vicious cycles: digital technologies and determinants of health in Australia. Health Promot Int.

[51] Banerjee A (2021). Digital health interventions and inequalities: the case for a new paradigm. BMJ Evid Based Med.

[52] Rich E, Miah A, Lewis S (2019). Is digital health care more equitable? The framing of health inequalities within England's digital health policy 2010-2017. Sociol Health Illn.

[53] Ahmed T, Rizvi SJ, Rasheed S, Iqbal M, Bhuiya A, Standing H, Bloom G, Waldman L (2020). Digital health and inequalities in access to health services in Bangladesh: mixed methods study. JMIR Mhealth Uhealth.

[54] Glied S, Lleras-Muney A (2008). Technological innovation and inequality in health. Demography.

[55] Fujioka JK, Budhwani S, Thomas-Jacques T, De Vera K, Challa P, Fuller K, Hogeveen S, Gordon D, Shahid S, Seto E, Shaw J (2020). Challenges and strategies for promoting health equity in virtual care: protocol for a scoping review of reviews. JMIR Res Protoc.

[56] Khilnani A, Schulz J, Robinson L (2020). The COVID-19 pandemic: new concerns and connections between eHealth and digital inequalities. J Inf Commun Ethics Soc.

[57] DeGuzman PB, Siegfried Z, Leimkuhler ME (2020). Evaluation of rural public libraries to address telemedicine inequities. Public Health Nurs.

[58] Gann B (2020). Combating digital health inequality in the time of coronavirus. J Consum Health Internet.

[59] Bommakanti KK, Smith LL, Liu L, Do D, Cuevas-Mota J, Collins K, Munoz F, Rodwell TC, Garfein RS (2020). Requiring smartphone ownership for mHealth interventions: who could be left out?. BMC Public Health.

[60] Weiss D, Sund ER, Freese J, Krokstad S (2020). The diffusion of innovative diabetes technologies as a fundamental cause of social inequalities in health. The Nord-Trøndelag Health Study, Norway. Sociol Health Illn.

[61] Karri K, Yarra P (2022). Inequities still exist in the use of digital health technology across different sociodemographic subgroups. Evid Based Nurs.

[62] Jaffe DH, Lee L, Huynh S, Haskell TP (2020). Health inequalities in the use of telehealth in the United States in the lens of COVID-19. Popul Health Manag.

[63] DeGuzman PB, Bernacchi V, Cupp CA, Dunn B, Ghamandi BJ, Hinton ID, Jameson MJ, Lewandowski DL, Sheffield C (2020). Beyond broadband: digital inclusion as a driver of inequities in access to rural cancer care. J Cancer Surviv.

[64] Sun N, Esom K, Dhaliwal M, Amon JJ (2020). Human rights and digital health technologies. Health Hum Rights.

[65] Ukoha EP, Feinglass J, Yee LM (2019). Disparities in electronic patient portal use in prenatal care: retrospective cohort study. J Med Internet Res.

[66] Hamideh D, Nebeker C (2020). The digital health landscape in addiction and substance use research: will digital health exacerbate or mitigate health inequities in vulnerable populations?. Curr Addict Rep.

[67] Erhunmwunsee L, Bhandari P, Sosa E, Sur M, Ituarte PH, Lui NS (2020). Socioeconomic, rural, and insurance-based inequities in robotic lung cancer resections. Video-Assist Thorac Surg.

[68] Ramírez AS, Estrada E, Ruiz A (2017). Mapping the health information landscape in a rural, culturally diverse region: implications for interventions to reduce information inequality. J Prim Prev.

[69] Matthews KA, Gallo LC (2011). Psychological perspectives on pathways linking socioeconomic status and physical health. Annu Rev Psychol.

[70] Norman CD, Skinner HA (2006). eHEALS: the eHealth literacy scale. J Med Internet Res.

[71] Torous J, Friedman R, Keshavan M (2014). Smartphone ownership and interest in mobile applications to monitor symptoms of mental health conditions. JMIR Mhealth Uhealth.

[72] Rogers EM (2010). Diffusion of innovation. 4th edition.

[73] Campos-Castillo C (2015). Revisiting the first-level digital divide in the United States: gender and race/ethnicity patterns, 2007-2012. Soc Sci Comput Rev.

[74] Schradie J (2012). The trend of class, race, and ethnicity in social media inequality: who still cannot afford to blog?. Inf Commun Soc.

[75] Link BG, Phelan J (1995). Social conditions as fundamental causes of disease. J Health Soc Behav.

[76] Tichenor PJ, Donohue GA, Olien CN (1970). Mass media flow and differential growth in knowledge. Public Opin Q.

[77] Mirowsky J, Ross CE (2003). Education, social status, and health.

[78] Kocher R, Emanuel EJ, DeParle NA (2010). The Affordable Care Act and the future of clinical medicine: the opportunities and challenges. Ann Intern Med.

[79] Kontos E, Blake KD, Chou WY, Prestin A (2014). Predictors of eHealth usage: insights on the digital divide from the Health Information National Trends Survey 2012. J Med Internet Res.

[80] Apter AJ (2014). Can patient portals reduce health disparities? A perspective from asthma. Ann Am Thorac Soc.

